# Early detection and treatment of cardiac dysfunction in cancer patients improve overall survival

**DOI:** 10.1590/1806-9282.20241437

**Published:** 2025-05-02

**Authors:** Lin Zheng, Jiawu Weng, Mingming Zhang

**Affiliations:** 1 Ningbo Fourth Hospital, Department of Cardiology – Ningbo, China.; 2 Ningbo Fourth Hospital, Department of Medical Oncology – Ningbo, China.

**Keywords:** Cancer, Heart failure, Quality of life, Cardiovascular diseases, Survival rate

## Abstract

**OBJECTIVE:**

The objective of this study was to analyze the impact of the early detection and treatment of cardiac dysfunction on overall survival in cancer patients.

**METHODS:**

A retrospective analysis was conducted on clinical data from 60 cancer patients with concurrent cardiac dysfunction, admitted between January 2020 and November 2022. Patients were divided into an early treatment group (n=35), where treatment began within 24 h of diagnosis, and a late treatment group (n=25), where treatment started after 24 h. The clinical efficacy, early diastolic filling velocity, fractional shortening, left ventricular ejection fraction, and Quality of Life Questionnaire-Core 30 scores were compared before and after treatment. Adverse reactions, cardiovascular events, and 1-year survival rates were also evaluated.

**RESULTS:**

The early treatment group showed higher total effective and survival rates compared to the late group (p<0.05). The post-treatment levels of early diastolic filling velocity, fractional shortening, left ventricular ejection fraction, and Quality of Life Questionnaire-Core 30 scores were statistically significantly higher in the early group (p<0.05), with no notable differences in adverse reactions (p>0.05). Kaplan-Meier analysis revealed a statistically significantly higher 1-year survival probability in the early treatment group (log-rank p=0.02).

**CONCLUSION:**

Early detection and treatment of cardiac dysfunction in cancer patients can improve treatment efficacy and survival rate, better improve cardiac function and quality of life, and reduce the occurrence of adverse cardiovascular events.

## INTRODUCTION

Cancer progression, characterized by abnormal cell proliferation, invasiveness, and metastasis, is a complex process influenced by factors, such as smoking, genetic predisposition, and environmental conditions^1^. Cancer treatments, including chemotherapy and radiotherapy, can cause cardiac dysfunction, worsening patients’ health and survival outcomes^2^. Cardiac dysfunction in cancer patients is defined by a reduction in left ventricular ejection fraction (LVEF), with a ≥10% decrease considered significant, as outlined by the American Society of Echocardiography and the European Association of Cardiovascular Imaging^3^. This condition often exacerbates patients’ health, emphasizing the need for early detection and treatment to improve survival rates^4,5^.

Cardiac dysfunction arises from the cardiotoxic effects of anticancer therapies. For instance, anthracyclines, commonly used in cancer treatment, are linked to dose-dependent cardiotoxicity, leading to left ventricular dysfunction and heart failure^6^. Additionally, targeted therapies, such as trastuzumab and tyrosine kinase inhibitors, can induce cardiac dysfunction^7^. Radiotherapy, especially when directed at the thoracic region, can also result in radiation-induced heart disease, contributing to conditions, such as myocardial fibrosis and coronary artery disease^8^.

The impact of cardiac dysfunction on cancer patients’ overall health is profound. A retrospective study found that pre-existing heart failure significantly increases mortality risk in breast cancer patients treated with trastuzumab. Furthermore, a systematic review by Lima et al. revealed a higher incidence of cardiac events and decreased survival in cancer patients who developed treatment-related cardiotoxicity^9^. These findings underscore the importance of early interventions to reduce the detrimental effects of cardiac dysfunction on cancer prognosis.

Early detection of cardiac dysfunction in cancer patients offers hope for improving survival by allowing timely interventions. LVEF assessments through echocardiography remain a key tool in monitoring cardiac function during cancer treatment. Emerging imaging techniques, such as global longitudinal strain (GLS) imaging, show promise in identifying early myocardial dysfunction, enabling interventions before irreversible damage occurs^9^. Additionally, cardiac biomarkers such as troponins and natriuretic peptides provide valuable insights for the early detection and management of cardiotoxicity^10^.

In summary, early detection and treatment of cardiac dysfunction in cancer patients are vital for enhancing survival and quality of life. A multidisciplinary approach integrating advanced imaging and biomarkers into clinical practice can improve early detection and guide interventions, thereby mitigating the impact of cardiac dysfunction on cancer outcomes. This study aims to evaluate the impact of the early detection and treatment of cancer-related cardiac dysfunction on clinical outcomes, particularly survival rates, cardiac function, and quality of life. Using echocardiography-based cardiac function assessments and validated quality-of-life measures, we compare the efficacy of initiating treatment within 24 h of diagnosis versus delayed treatment. By identifying the benefits of timely interventions, we seek to provide evidence supporting early clinical management to improve the prognosis of cancer patients with cardiac dysfunction.

## METHODS

### Study population

In this retrospective analysis, the clinical data of 60 cancer patients with concurrent cardiac dysfunction admitted to our hospital from January 2020 to November 2022 were reviewed. Based on the time of diagnosis and initiation of treatment for cardiac dysfunction, the patients were divided into an early treatment group (diagnosis and treatment of cardiac dysfunction initiated within ≤24 h) consisting of 35 cases and a late treatment group (diagnosis and treatment initiated after >24 h of diagnosing cardiac dysfunction) consisting of 25 cases. The inclusion criteria were as follows: (1) confirmed diagnosis of cancer by histopathological examination; (2) absence of skin infection at the site of ultrasound probe placement and diagnosis of cardiac dysfunction by echocardiography; (3) age ≥18 years; and (4) availability of complete clinical data. The exclusion criteria were: (1) a history of previous cardiac surgery; (2) existence of cognitive impairment, psychiatric disorders, or non-compliance; and (3) pregnancy or lactation in females.

### Intervention methods

Patients in the early treatment group received treatment within 24 h of diagnosing cardiac dysfunction, while the late group received treatment after 24 h. Cardiac dysfunction was detected via echocardiography using the Philips EPIQ7C Doppler ultrasound. Key parameters measured included early diastolic filling velocity (E), fractional shortening (FS), and left ventricular ejection fraction (LVEF), with a ≥,0% LVEF decrease indicating dysfunction. The patients were treated with sacubitril/valsartan tablets (Beijing Novartis), starting at 50 mg twice daily, increased to 100 mg after 2 weeks, and adjusted based on blood pressure and cardiac function, up to 200 mg, for a total of 10 weeks.

### Outcome measures

1. Baseline characteristics: These include gender, age, body mass index (BMI), cancer type, and other relevant data for both groups^6^.
2. Clinical efficacy: Marked improvement was defined as the restoration of LVEF to baseline levels, while improvement indicated partial LVEF recovery. The total effective rate was calculated as (marked improvement+improvement)/total cases×100%.
3. Cardiac function indicators: The pre- and post-treatment levels of E, FS, and LVEF were measured.
4. Quality of life: This was assessed using the Quality of Life Questionnaire-Core 30 (QLQ-C30), covering physical, role, emotional, cognitive, and social functioning, with scores out of 100^7^.
5. Adverse reactions: These include monitored occurrences of nausea, vomiting, headache, renal impairment, edema, and hypotension.
6. Adverse cardiovascular events: These include tracked arrhythmia, angina, heart failure, and cardiogenic shock over 1 year.
7. Survival rate: This was calculated as survivors/total cases×100% after 1 year.

### Statistical analysis

Data were analyzed using SPSS 25.0 (IBM, Armonk, NY, USA). Categorical data were expressed as n (%). For sample sizes ≥40 and number of categories (t)≥5, the chi-square test was applied. For t between 1 and 5, a chi-square test with correction was used, and for t<1 or sample sizes <40, the Fisher's exact test was employed. Continuous variables were presented as mean±standard deviation and analyzed using the t-test, with p<0.05 being considered statistically significant.

## RESULTS

### Comparison of baseline data between the two groups

As shown in Table 1, the early treatment group included 24 males and 11 females, with an age range of 50–72 years (mean±standard deviation: 61.38±5.40 years) and a BMI of 17.1–24.5 kg/m² (mean±standard deviation: 20.48±3.26). The late treatment group consisted of 15 males and 10 females, with an age range of 52–75 years (mean±standard deviation: 61.60±5.32 years) and a BMI of 16.5–24.0 kg/m² (mean±standard deviation: 20.25±3.58). Tumor types were similar across both groups, including liver, gastric, lung, breast, and colorectal cancers. No statistically significant differences in gender, age, BMI, or tumor types were observed between the groups (p>0.05), indicating that they were comparable.

**Table 1:** Comparison of baseline data between the two groups.

Group	Gender		Age (years)	BMI (kg/m^2^)	Tumor types				
	Male	Female			Hepatocellular carcinoma	Gastric cancer	Lung cancer	Breast cancer	Colorectal cancer
Early treatment group (n=35)	24 (68.57)	11 (31.43)	61.38±5.40	20.48±3.26	9 (25.71)	7 (20.00)	8 (22.86)	5 (14.29)	6 (17.14)
Late treatment group (n=25)	15 (60.00)	10 (40.00)	61.60±5.32	20.25±3.58	6 (24.00)	5 (20.00)	6 (24.00)	5 (20.00)	3 (15.00)
**χ** ^2^/t	0.471		0.157	0.259	0.277				
p	0.493		0.876	0.797	0.991				

BMI: body mass index.

### Comparison of clinical efficacy between the two groups

In the early treatment group, 23 patients showed marked improvement, 10 patients showed improvement, and 2 patients did not show improvement, resulting in a total effective rate of 94.29%. In the late treatment group, 10 patients experienced marked improvement, 8 showed improvement, and 7 were classified as ineffective, giving a total effective rate of 72.00%. The total effective rate in the early treatment group was statistically significantly higher than that in the late treatment group (p<0.05), indicating that the early detection and treatment of cardiac dysfunction in cancer patients can lead to better treatment outcomes.

### Comparison of cardiac function indicators before and after treatment between the two groups

As shown in Table 2, there were no statistically significant differences in the levels of E, FS, and LVEF before treatment between the two groups (p>0.05). However, the post-treatment levels of E, FS, and LVEF were statistically significantly higher in the early treatment group than those in the late treatment group (p=0.019, 0.006, and 0.013, respectively), indicating that the early detection and treatment of cardiac dysfunction in cancer patients can better improve their cardiac function.

**Table 2:** Comparison of cardiac function indicators before and after treatment in the two groups.

Group	E (cm/s)		FS (%)		LVEF (%)	
	Before treatment	After treatment	Before treatment	After treatment	Before treatment	After treatment
Early treatment group (n=35)	68.56±3.12	74.40±3.25	32.80±4.02	37.75±4.14	62.54±5.72	67.75±4.32
Late treatment group (n=25)	69.10±3.20	72.38±3.14	32.56±4.28	34.68±4.06	61.90±5.88	64.80±4.46
t	0.654	2.407	0.222	2.855	0.422	2.573
p	0.516	0.019	0.825	0.006	0.674	0.013

E: early diastolic filling velocity, FS: fractional shortening, LVEF: left ventricular ejection fraction.

### Comparison of Quality of Life Questionnaire-Core 30 scores before and after treatment between the two groups

As depicted in Table 3, there were no statistically significant differences in the QLQ-C30 scores for physical, role, emotional, cognitive, and social functioning before treatment between the two groups (p>0.05). However, the post-treatment QLQ-C30 scores of physical, role, emotional, cognitive, and social functioning were statistically significantly higher in the early treatment group than those in the late treatment group (p=0.001, 0.001, 0.001, 0.003, and 0.005, respectively), suggesting that the early detection and treatment of cardiac dysfunction in cancer patients can better enhance their quality of life.

**Table 3:** Comparison of Quality of Life Questionnaire-Core 30 scores before and after treatment in the two groups.

Group	Physical function		Role function		Emotional function		Cognitive function		Social function	
	Before treatment	After treatment	Before treatment	After treatment	Before treatment	After treatment	Before treatment	After treatment	Before treatment	After treatment
Early treatment group (n=35)	53.86±5.32	65.74±4.25	51.32±5.28	66.47±6.04	53.32±5.80	64.35±5.76	57.36±6.42	69.74±5.96	60.32±5.50	74.42±5.84
Late treatment group (n=25)	53.50±5.48	60.90±4.56	50.86±5.35	58.75±5.98	53.54±5.95	57.90±6.04	58.20±6.56	64.85±5.86	61.02±5.98	69.90±5.95
t	0.255	4.219	0.331	4.901	0.143	4.191	0.495	3.155	0.469	2.933
p	0.799	0.001	0.742	0.001	0.887	0.001	0.622	0.003	0.641	0.005

### Comparison of adverse reactions between the two groups

In the early treatment group, 8.57% of the patients experienced renal impairment, 8.57% had vasogenic edema, and 8.57% had hypotension. In the late treatment group, 8.00% of patients experienced nausea and vomiting and 8.00% had headaches and dizziness. There were no statistically significant differences in the occurrence of adverse reactions between the two groups (p>0.05), suggesting that the early detection and treatment of cardiac dysfunction in cancer patients does not increase the risk of adverse reactions, thereby demonstrating a similar safety profile between the groups.

### Comparison of adverse cardiovascular events between the two groups

In the early treatment group, 5.71% of the patients experienced arrhythmia and angina. In contrast, the late treatment group had a higher occurrence of adverse cardiovascular events, with 28.00% of the patients experiencing arrhythmia, 16.00% experiencing angina, and 8.00% developing heart failure. The overall occurrence rate of adverse cardiovascular events was statistically significantly lower in the early treatment group compared to the late treatment group (p<0.05), indicating that the early detection and treatment of cardiac dysfunction in cancer patients can effectively reduce the risk of adverse cardiovascular events.

### Comparison of survival rates between the two groups

A 1-year follow-up revealed a survival rate of 91.43% in the early treatment group, compared to 68.00% in the late treatment group. The survival rate in the early treatment group was statistically significantly higher than that in the late treatment group (p<0.05), suggesting that the early detection and treatment of cardiac dysfunction in cancer patients can significantly improve their survival rate.

## DISCUSSION

The incidence of malignant tumors is rising in our country. While modern anti-tumor therapies have extended survival for cancer patients, they also introduce cardiac toxicity. Overall, the benefits of these treatments outweigh the risks^8^. Understanding cardiac toxicity mechanisms and minimizing high-risk factors are crucial for effective management^9^. The early detection of cardiac toxicity has gained increasing attention, with a focus on reducing long-term complications^10^. Cancer treatment-induced cardiac dysfunction is a major cause of morbidity and mortality, highlighting the importance of early detection and intervention to improve patient outcomes^11^.

Early interventions offer distinct advantages by mitigating the progression of cardiotoxicity at an early stage, preventing irreversible myocardial damage^12^. This study similarly concludes that the early detection and treatment of cardiac dysfunction improves cardiac function and clinical outcomes in cancer patients. Sacubitril/valsartan inhibits neprilysin and blocks angiotensin II receptors, counteracting the renin–angiotensin–aldosterone system, delaying ventricular remodeling, and alleviating cardiac toxicity^13^. Administering sacubitril/valsartan within 24 h of diagnosis can reverse cardiac dysfunction and prevent deterioration. Onishi et al.^14^ also found that the early evaluation and treatment of cancer-related cardiac dysfunction improves patients’ quality of life, as early intervention lowers neprilysin, inhibits natriuretic peptide degradation, reduces aldosterone levels and blood pressure, promotes diuresis and vasodilation, and prevents myocardial remodeling. Sacubitril's antioxidant and anti-inflammatory properties further alleviate sodium retention and improve exercise tolerance, enhancing the quality of life. Early interventions may reduce systemic inflammation and endothelial dysfunction caused by cancer therapies, both of which are linked to adverse cardiac outcomes. By maintaining vascular homeostasis and improving coronary blood flow, early treatment reduces the incidence of adverse cardiovascular events, such as arrhythmias and myocardial infarction.

Khoury et al.^15^ found that the early treatment of cardiac dysfunction in cancer patients improves prognosis and survival rates by preventing the deterioration of cardiac function and reducing cardiovascular events. This study supports the conclusion that the early detection and treatment of cardiac dysfunction lowers the risk of adverse cardiovascular events. Importantly, no statistically significant difference in the incidence of adverse reactions between the early and late treatment groups was found (p>0.05), indicating that early treatment is safe and does not increase the risk of side effects.

In summary, the early detection and treatment of cardiac dysfunction in cancer patients can improve their effectiveness and survival rate, better enhance their cardiac function and quality of life, and reduce the occurrence of adverse cardiovascular events. First, the small sample size and single-center design in this study may limit the generalizability of the findings. Future research should include multi-centric, large-scale prospective studies to validate the observed benefits of early detection and treatment in broader patient populations. Such studies could employ randomized controlled trial designs to minimize potential biases and enhance the strength of the evidence. Second, the relatively short follow-up period restricts insights into the long-term impact of early interventions on survival and cardiac function. Extended follow-up periods, combined with longitudinal assessments, are recommended to better evaluate sustained outcomes and delayed adverse effects. Additionally, the reliance on echocardiographic parameters such as LVEF and GLS, while effective, could be supplemented with advanced imaging modalities like cardiac magnetic resonance to provide more comprehensive assessments of myocardial health. Incorporating biomarkers such as high-sensitivity troponins and natriuretic peptides may further improve the early detection and risk stratification of cardiac dysfunction in cancer patients.
